# Nutritionists as policy advocates: the case of obesity prevention in Quebec, Canada

**DOI:** 10.1017/S1368980021004997

**Published:** 2022-07

**Authors:** Jacqueline Wassef, François Champagne, Lambert Farand

**Affiliations:** University of Montreal, School of Public Health, Department of Health Management, Evaluation and Policy, C.P. 6128, Downtown campus, Montreal, QC H3C 3J7, Canada

**Keywords:** Policy making, Advocacy, Nutritionist, Obesity, Public health nutrition, Canada

## Abstract

**Objective:**

A core function of the public health nutrition workforce is advocacy. Little is known of the nutritionists’ role in policymaking from a policy process theory perspective. The current study analyses the nutritionists’ role in advocating for a six-year governmental plan on obesity prevention in Quebec, Canada.

**Design:**

We conducted qualitative research using Quebec’s obesity policy as a case study to understand the role of nutritionists in advocating for obesity prevention policies. A conceptual framework combining the Advocacy Coalition Framework with a political analysis model based on the Theory of the Strategic Actor was developed to analyse the beliefs, interests and strategies of policy actors including nutritionists. Data sources comprised semi-structured open-ended interviews with key policy actors (*n* 25), including eight nutritionists (32 %) and policy-related documents (*n* 267). Data analysis involved thematic coding and analysis using NVivo 11 Pro.

**Setting:**

Quebec, Canada.

**Participants:**

Key policy actors including nutritionists.

**Results:**

Nutritionists formed the core of the dominant public health coalition. They advocated for an inter-sectoral governmental plan to prevent obesity through enabling environments. Their advocacy, developed through an iterative process, comprised creating a think tank and reinforcing partnerships with key policy actors, conducting research and developing evidence, communicating policy positions and advocacy materials, participating in deliberative forums and negotiating an agreement with other coalitions in the policy subsystem.

**Conclusions:**

Nutritionists’ advocacy influenced agenda setting and policy formulation. This research may contribute to empowering the public health nutrition workforce and strengthening its advocacy practices. It informs practitioners and researchers concerned with obesity policy and workforce development.

A core function of the public health nutrition workforce is advocacy^(1–6)^. Advocacy occurs in dynamic and complex socio-political environments^(7)^. Being part of a complex policymaking process, advocacy practices require a thorough understanding of policymaking to identify leverage points and influence key actors^(1,8)^. Research suggests that the current public health nutrition work force is disengaged and unprepared for advocacy^(1,9–12)^. Advocating for structural and political responses to improve macro-level environmental factors influencing what people eat is quite challenging^(1)^. The complexity of the policy issue, the diversity of actors, the entrenched beliefs’ dichotomies and food industry lobbying among others complicate the policy process^(13,14)^.

In 2006, the Ministry of Health and Social Services (MOH) in the Province of Quebec, Canada, issued the ‘Governmental Action Plan for the Promotion of Healthy Lifestyles and the Prevention of Weight-Related Problems 2006–2012, Invest in the Future’ (governmental action plan (GAP))^(15)^. Its goal was to improve Quebeckers’ quality of life through the provision of living environments that promote the adoption and maintenance of healthy lifestyles^(15)^. The GAP consisted of five intervention axes – promoting a healthy diet, fostering a physically active lifestyle, enabling social norms, promoting research and knowledge transfer and improving services for people with weight problems^(15)^. The plan comprised seventy-five actions to be implemented by ten participating ministries and government agencies (Table 1). A public–private partnership (PPP) was negotiated with a well-established philanthropic organisation in Canada, the Lucie and André Chagnon Foundation to support GAP-related community interventions. Shortly after the GAP adoption, the National Assembly of Quebec enacted a law instituting the Healthy Lifestyles Fund, a 400-million-dollar bipartite fund^(16,17)^ which operated until 2019^(18)^. Nutritionists participated significantly in the policy process from agenda setting to termination and evaluation.

**Table 1:** GAP participating ministries/institutions – Quebec, Canada

(1) Ministry of Agriculture, Fisheries and Food
(2) Ministry of Education, Leisure and Sports
(3) Ministry of Family, Elderly and Women’s Condition
(4) Ministry of Health and Social Services
(5) Ministry of Justice – Consumer Protection Office
(6) Ministry of Labor and Social Solidarity
(7) Ministry of Municipal Affairs and Regions
(8) Ministry of Transportation
(9) National Public Health Institute
(10) Youth Secretariat

Public policy action to address nutrition outcomes among high-income countries has achieved limited progress and is still inconsistent^(1,19)^. Generally, health advocates have a limited understanding of the policy process that translates to a reduced capacity to identify leverage points, influence policymakers and gain support^(9,20–22)^. Policy-focused studies tend to describe the content of the policy or evaluate its impact rather than bring insights to the policymaking process^(1,23,24)^. The use of policy process theories drawn from political science to analyse and improve the nutrition^(1)^ and the obesity-policy processes^(8)^ is still in its early stages. Using a conceptual framework drawn from political science, the current study aims to explain the role of nutritionists in the GAP agenda setting and formulation through an analysis of their belief system characteristics, networks and advocacy strategies. To the best of our knowledge, this is the first study analysing nutritionists’ advocacy for a governmental obesity policy.

## Methodology

**Fig. 1:**
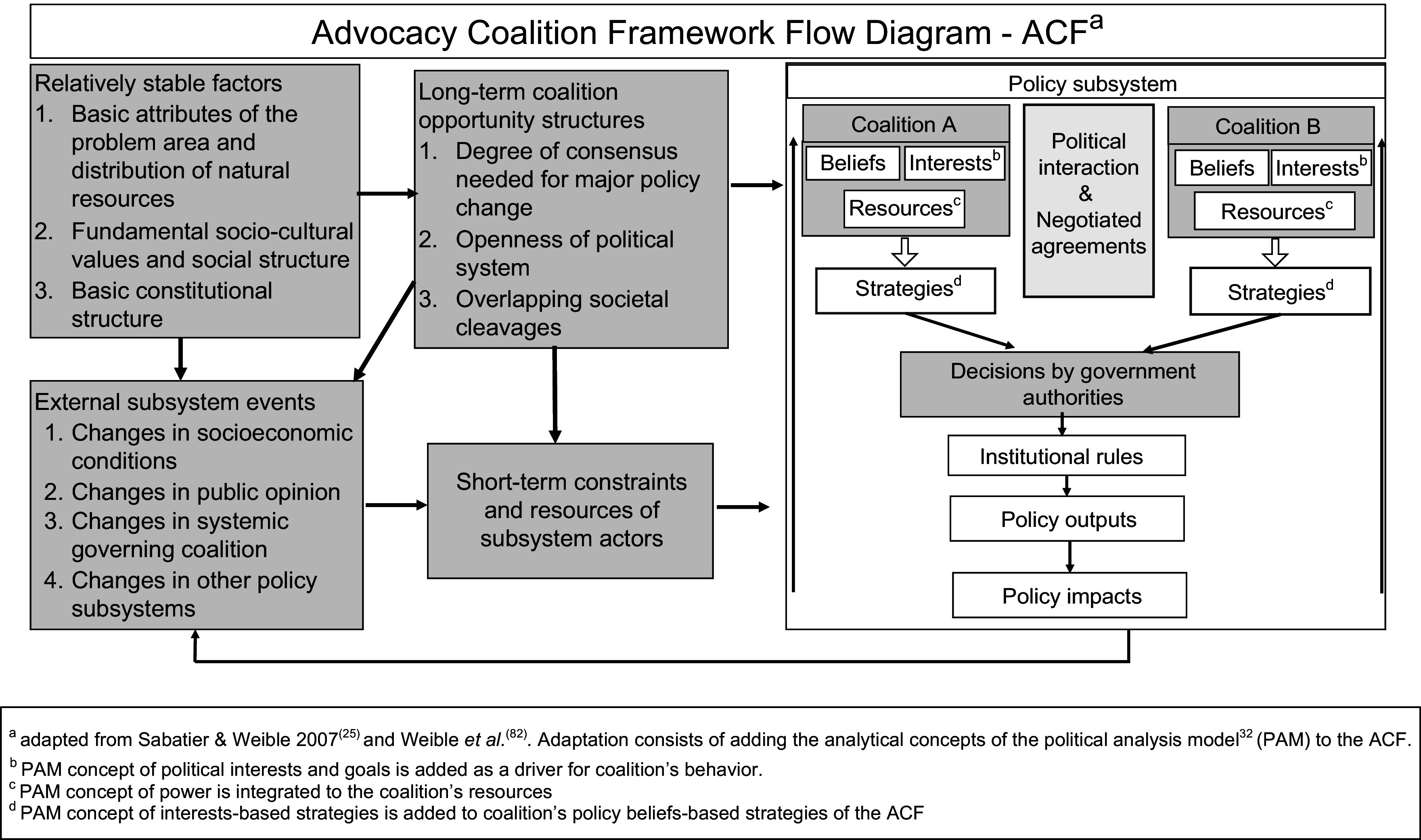
Conceptual Framework

A case study research design was used to answer the research questions (Panel 1). The case is the agenda setting and formulation of the GAP by the Province of Quebec. We used the Advocacy Coalition Framework (ACF)^(25)^ to identify advocacy coalitions and explain the GAP’s processes for agenda setting and formulation (Fig. 1). The ACF outlines a three-tiered belief system comprising deep core, policy core and secondary beliefs and groups actors who share the same policy core beliefs and coordinate substantially in a coalition^(26–28)^. While shared beliefs bind actors within a coalition, policy core beliefs are the most relevant for coalition formation because they entail normative and empirical commitments that are salient to one policy domain^(28,29)^. Coalitions within a policy subsystem marshal resources and advance strategies to translate their beliefs into policies. The policy subsystem is embedded in a larger context and is influenced by factors that can be dynamic or stable and that favour or constrain the coalitions’ strategies^(30)^ (Fig. 1).

Panel I. Methodology

**Research question**

What is the role of nutritionists in the advocacy for the agenda setting and formulation of the GAP?

**Study Design and Conceptual Framework**

- Single case-study design
- Conceptual framework combining:
  - Advocacy Coalition Framework
  - Political Analysis Model

**Data sources**

- Semi-structured interviews with key informants
  - *n* 25 of which 8 were nutritionists (see Table 2 for the demographics of key informants & nutritionists)
  - Duration of interviews with nutritionists ranged between 66 and 190 min
  - May 2016 – September 2017
  - Detailed interview guide translated into French to suit the context of Quebec (Table 1 – Supplementary material)
  - Strategic sampling and snowballing technique
- Policy-related documents (*n* 267)

**Data analysis**

- Interview transcription using Dragon software
- Thematic coding based on a coding guide (available upon request)
- Sabatier’s three-tier belief structure:
  - Deep core
  - Policy core
  - Secondary aspects
- NVivo 11 Pro

The ACF assumes that actors are instrumentally rational and that they pursue policy-oriented goals rather than self-interests of economic or political order^(28)^. Many would disagree with this assumption and would believe that self-interests, or power maximisation, are major drivers of political decision making^(29)^. So, Sabatier’s altered belief-based explanation is insufficient to explain policy change^(29)^. An interest-based explanation (favouring conditions that ensure survival, growth and autonomy of the actor) complements a belief-based one, and it may reveal gaps between interests and policy core beliefs^(29,31)^.

Therefore, a political analysis model was integrated into the ACF to analyse the political interests and goals of policy actors, which are less emphasised in the ACF^(32)^. The model is inspired by the Theory of the Strategic Actor of Crozier and Friedberg^(33)^ which stipulates that, within a given structural and political context where an innovation is being planned and implemented, actors follow their individual goals. Concurrently, the innovation has its own goals, namely ‘the theoretically defined potential for change,’ associated with it^(32) (p.107)^. The actors’ support for the innovation depends on how congruent its goals are with theirs and the extent to which it helps them achieve their own. Actors’ support or opposition manifests as instrumental strategies interacting within an environment characterised by a preexisting distribution of power among actors. Dominant actors with relatively more power are more likely to attain their goals^(32)^.

### Data sources

The first data source consisted of semi-structured interviews of key informants (*n* 25). An interview guide was developed based on the concepts of the ACF and the political analysis model. A purposive snowball sampling strategy was used to recruit key informants, and data saturation determined the final sample size. Key informants were selected based on their participation in the GAP workgroups and their contribution to the GAP either through advocacy, formulation, implementation, or evaluation. A key policy actor who was the national GAP coordinator (2007–2012) and a member of the think tank, *Provincial Workgroup on Weight-Related Problems* in Quebec (PWG), validated the key informants’ list. Sample variability based on the relevance of the key informants to the research questions guided the strategic sampling. The ministerial/organisational and the workgroup affiliation of the key informant were considered in the purposeful sampling; we interviewed key informants from all major ministries/organisations and workgroups contributing to or formulating the GAP (Table 2).

**Table 2:** Key informants’ and nutritionists’ demographics

	Key Informants (*n* 25)	Nutritionists (*n* 8; 32 %)
Organisation*/Ministry		
Academic institution	2	2
Association of Directors of Public Health	1	–
Association for Public Health of Quebec	3	2
Institute of Nutrition and Functional Foods	1	1
Media	1	–
Ministry of Agriculture, Fisheries and Food	1	–
Ministry of Education, Leisure and Sports	6	–
Ministry of Health and Social services	5	2
Ministry of Transportation	1	–
National Public Health Institute	4	2
NGO (EquiLibre, Québec Council on Weight and Health)	2	2
Private sector (health services and sport facilities)	1	–
Order of Dietitians-Nutritionists of Quebec	1	1
Québec en forme and Lucie and André Chagnon Foundation	3	–
Regional Public Health Directorate	1	–
Sports Québec	1	–
Workgroup†		
Provincial Workgroup on Weight-Related Problems	7	5
Provincial Task Force on Prevention in Youth (members, direction and collaborators)	9	3
GAP (authors, writing directors and collaborators)	12	3

NGO, non-governmental organisation; GAP, governmental action plan.

* Some key informants had more than one affiliation during the GAP formulation.

† Some key informants were members of more than one workgroup.

Nutritionists formed a subsample of key informants (*n* 8; 32 %) and included those practicing in public health at the provincial, regional and local levels. Some of them participated significantly in the complete policy cycle, from agenda setting to evaluation and termination (*n* 2), including the national GAP coordinator. The majority (*n* 5) were members of the PWG. Interviews with all study participants were recorded following their approval. Informants were deidentified, except those who allowed their identity to be linked to their statements. Table 2 presents key informants’ and nutritionists’ demographics by institutional affiliation and workgroup.

A document database (*n* 267) was built using three sources: organisational and ministerial documents; briefs, bills, committee reports and other documents of the National Assembly of Quebec and unpublished reports, meeting minutes and other documents provided by key informants. We reviewed all position papers, policies, guidelines, press releases and scientific opinions on obesity prevention from the concerned ministries and organisations, the National Assembly of Quebec, professional regulatory bodies, academic institutions and private sector organisations. For data triangulation, we reviewed other documents, such as annual reports and strategic plans, including those of the MOH, Quebec en Forme, Lucie and André Chagnon Foundation, Sports and Leisure Secretariat, Ministry of Agriculture, Fisheries and Food and the Order of Dietitians-Nutritionists of Quebec (ODNQ). We looked for convergence of various sources of information across different data sources to corroborate our research findings increasing as such their credibility. We also looked for disconfirming evidence that would support rival explanations. An additional technique used to increase the credibility of our research findings is the member checking technique^(34)^. Some nutritionists, selected based on their depth of involvement in and knowledge of the GAP development, were asked to express their comments on a draft version of this article.

### Data analysis

Interviews of key informants, including nutritionists, were transcribed using Dragon software. NVivo 11 Pro was used to aid analysis of verbatim transcripts. A coding guide was developed based on the conceptual framework. We used thematic codes to analyse internal and external events, actors’ beliefs, resources, goals, strategies, opportunities and constraints. We performed the current analysis on all our data sets including nutritionists and other policy actors in the policy subsystem. Subthemes under each category emerged, and coding was performed iteratively to allow inclusion of earlier findings under emerging themes. To identify coalitions, we coded actors’ beliefs based on Sabatier’s three-tiered belief structure^(27,28)^. Deep core beliefs span all policy subsystems and refer to the ‘fundamental normative and ontological axioms’^(28)^. Policy core beliefs relate to one entire policy subsystem and include normative precepts on basic value priorities and groups whose welfare is of utmost concern. Precepts with a significant empirical component include beliefs on the seriousness of the problem, causal assumptions, policy preferences and priority policy instruments, levels of government qualified to deal with the problem and the desired distribution of authority between the market and the government^(27,28)^. Secondary beliefs are generally restricted to parts of the policy subsystem, such as the seriousness of some aspects of the problem in specific settings^(27,28)^.

To analyse actors’ strategies, we created a semantic structure connecting the concepts: (i) actors concerned; (ii) actors’ coalition; (iii) actors’ goals; (iv) actors’ strategies related to their political interests and (v) actors’ strategies related to their policy beliefs. Strategies pertaining to the ACF included organising think tanks; funding research to develop evidence; recruiting allies in positions of formal legal authority; utilising information and evidence to solidify own membership, negotiate with opponents, convince policymakers and sway public opinion; communicating policy positions and launching lobbying campaigns and garnering public support^(25)^. Following the analysis of the coalitions’ political goals, we coded for strategies that helped the coalitions achieve those goals.

Following the initial analysis, the thematic codes pertaining to nutritionists were extracted from the coding pool of key informants and reanalysed. Themes related to the nutritionists’ beliefs and the policy issue were reanalysed to identify the internal policy framing within their policy community. Framing echoes the processes of interpreting and communicating issues through social discourse and interaction^(35)^. Internal policy framing reflects the understanding and portrayal of the issue by the policy community^(36,37)^. The semantic structure created to analyse actors’ political and belief-oriented strategies was reanalysed for nutritionists.

The analytic technique we used for our case study is explanation building^(38)^. This procedure consists of analysing the data by building an explanation about the case^(38)^. It is a similar analytic technique to process tracing in political science research and is a type of pattern matching^(38)^. The assumption that informed the analysis is based on our conceptual framework (Fig. 1) whereby a coalition, in this case that of public health formed and spearheaded by nutritionists, advances strategies based on their beliefs and political interests to propose policies and have them adopted. Our assumption was that the GAP was the outcome of a negotiated agreement between coalitions and that nutritionists played a key role in negotiating its content. Explanation building was iterative, starting with the initial assumption and looking for other plausible explanations supported by the data.

## Results

### Advocacy coalitions’ policy beliefs

We identified four advocacy coalitions^(27,28)^ interacting in the policy subsystem and named them based on a concise descriptor of their policy beliefs: an *Enabling Environments Advocacy Coalition (EEAC)*, a *Healthy Lifestyles Promoting Advocacy Coalition (HLPAC)*, an *Agri-Food Advocacy Coalition (AFAC)* and a *Community Development Advocacy Coalition (CDAC)*. We provide illustrative quotes^§^ in Table 2 (online Supplementary Material). Members of the EEAC were public health actors from government and non-government sectors including the MOH, the National Public Health Institute, the Association for Public Health of Quebec and the PWG. Nutritionists constituted the core of the EEAC, the dominant advocacy coalition. Nutritionists believed that obesity was an epidemic that the government must address urgently. At the core of their beliefs was a concern related to the severity of the increased burden of chronic diseases and health care expenses. Obesity, being overweight and excessive concern with weight and body image were part of a multi-faceted weight problem^(39–41)^ whose fundamental causes were obesogenic environments and those promoting unique beauty models. They believed that, under the leadership of the health sector, governments must adopt policies to create enabling environments so that healthy choices became the easy ones.^§1,26(40)^ Although focusing on children was a good political strategy that resonated well with policymakers, nutritionists believed interventions should address all population groups. They also believed that the private sector had to participate in this process, through compulsory measures if necessary. Table 3 summarises the core policy beliefs of nutritionists.

**Table 3:** Core policy beliefs of nutritionists*

Policy core belief components†	Nutritionists’ beliefs
Seriousness of the problem	Obesity is a public health problem, an epidemic.
	Unhealthy lifestyles are a public health problem.
	Weight problems include overweight, obesity and excessive concern with weight and body image.
	Urgent governmental action is needed to address weight problems.
Basic causes	Only environmental causes can explain the steep rise in obesity over the last decades and the excessive concern with weight.
	Biologic/individual causes and behavioural causes, although important, cannot be blamed for the obesity epidemic.
Responsibility for action	Greater emphasis on collective and government responsibility for action as compared with individual responsibility.
Priority group	Priority to the population as a whole, rather than to specific groups such as children and youth.
Policy preferences	Policy solutions should be designed to create enabling environments, namely the social, physical, political and economic environments.
	Behavioural solutions are still needed only when enabling environments are in place.
	Health services solution should be well designed and available when needed. This solution should be accompanied with effective preventive measures.
Distribution of authority	Authority should be distributed between national, regional and local levels.
	Provincial preventive actions should guide regional and local ones.
Private sector role	Is a key player in improving environments. Incentives are not enough, compulsory measures may be required (e.g. taxation, marketing and advertising regulations including advertising to children and marketing of the weight loss industry).
Inter-sectoral collaboration	Every sector has a significant role in creating enabling environments.

* Nutritionists were part of the Enabling Environments Advocacy Coalition, the dominant advocacy coalition in the Obesity Prevention Policy Subsystem – Quebec, Canada

† Related to weight problems and healthy lifestyles; healthy lifestyles include a healthy diet and a physically active lifestyle; weight problems include overweight, obesity and excessive concern with weight

The AFAC was concerned with the autonomy and competitiveness of the agri-food sector, freedom of choice and individual responsibility. While they believed that unhealthy lifestyles were the leading cause of obesity, they considered that the choice whether to exercise and consume a healthy diet or not was an individual one. The solutions lay in increasing individuals’ awareness,^§2^ thus safeguarding the purchasing power, market rules and competitiveness of the agri-food sector. Therefore, the AFAC believed that adherence to any new governmental measure should be voluntary. Members of the AFAC were from the Ministry of Agriculture, Fisheries and Food and the associations of the Agri-Food sector.

The third coalition was the *Healthy Lifestyles Promoting Advocacy Coalition*. They believed that obesity was the result of unhealthy lifestyles, so the solution would lay in promoting healthy lifestyles for general health and well-being and not solely for obesity prevention.^§3^ They warned that healthy lifestyles, and particularly physical activity, should not be promoted solely to reduce obesity and the risk of chronic diseases; instead, positive messages promoting well-being and pleasure should be emphasised. This coalition believed that intrinsic motivation rather than the fear of obesity should drive behaviour change and that this message should be reinforced through social marketing campaigns to improve social norms. Members of the HLPAC were from the Ministry of Education, the Sport and Leisure Secretariat and its partners and the provincial physical activity program *Kino-Québec*. Launched in 1978 by the government of Quebec, Kino-Québec is designed to encourage active living to improve people’s well-being^(42)^.

The *Community Development Advocacy Coalition* believed that unhealthy lifestyles have devastating health effects, including obesity but that determining the causes and solutions depended on community diagnosis and needs.^§4^ The CDAC believed in the need for an increased government investment in children’s health. Therefore, they promoted a bipartite partnership with the government to fund preventive actions. Members were from the philanthropic organisation *Lucie and André Chagnon Foundation* and its affiliated organisation *Québec en forme*. The Foundation had been active in Quebec since 2000^(43)^. As a result of an expert consultation launched in 2001, the Foundation decided to work on prevention of disease and poverty in children^(44)^ and prioritised sports and physical activity^(45)^. Québec en Forme, a not-for-profit organisation, was created in 2002 through a PPP between the Foundation and the Government of Quebec. Their four-year project would improve the overall health and autonomy of 4- to 12-year-old children in disadvantaged communities through a programme that integrates physical activity and sport into their lifestyles^(45)^.

### Strategies

**Fig. 2:**
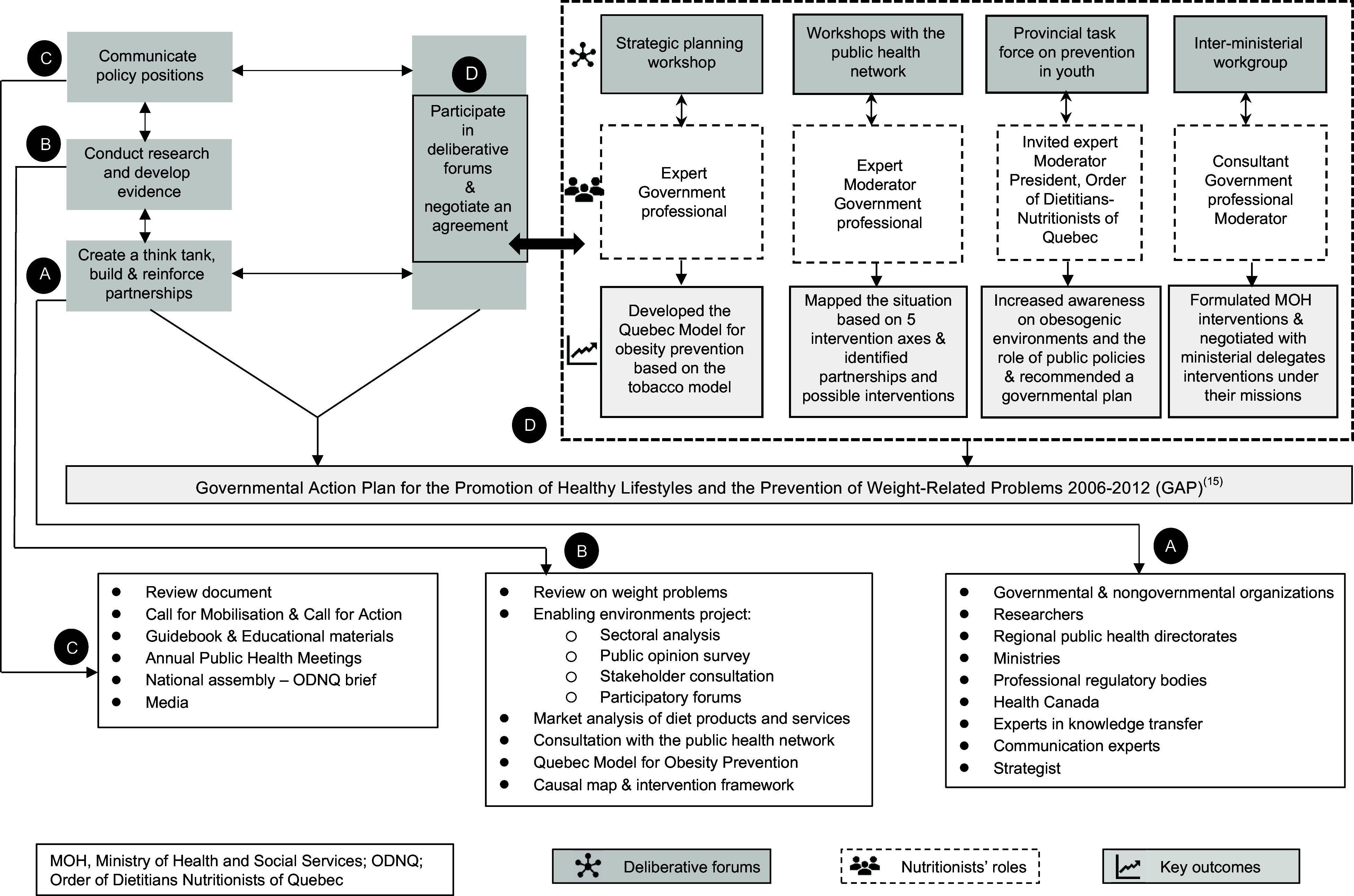
Advocacy Strategies of Nutritionists and their Key Outcomes Contributing to the Governmental Action Plan **(**GAP)

The nutritionists’ advocacy strategies developed through an iterative and comprehensive process that included building and reinforcing strategic partnerships, developing evidence, communicating policy positions and advocacy materials, participating in deliberative forums and negotiating an agreement. The main strategies under each component are described hereinafter. Figure 2 summarises these strategies and their key outcomes contributing to the GAP.

#### Build and reinforce strategic partnerships

Nutritionists working on obesity prevention created a cohesive and organised think tank, the PWG, under the auspices of the Association for Public Health of Quebec in the year 2000^(40,41)^ with the concept of enabling environments for health at its core. The PWG reached a consensus on the problem definition, its solutions and the urgent need for governmental action on obesogenic environments.^§5^ Mostly composed of nutritionists, the PWG formed the backbone of the EEAC. Previously established networks and partnerships helped recruit members (Table 3 – online Supplementary Material)^(40)^. A key ally was the national public health director (NPHD) who (a) shared the nutritionists’ beliefs regarding the framing of the problem and (b) rallied around the cause.^§6^

**Fig. 3:**
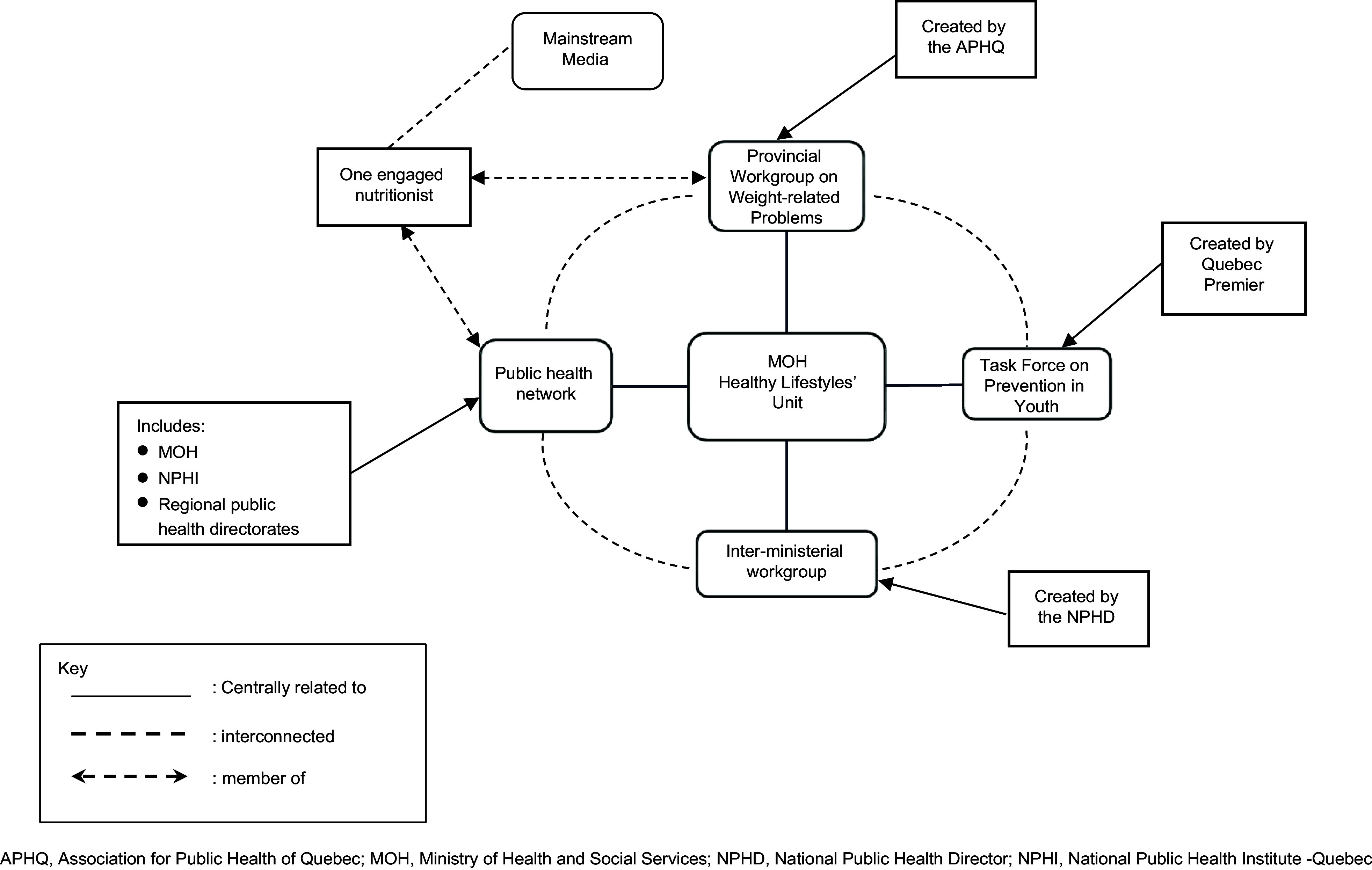
Nutritionists’ Advocacy Network – Quebec, Canada

Within the EEAC, the nutritionists had a highly structured network with five coordination channels, as illustrated in Fig. 3. Central to these channels was the Healthy Lifestyles Unit at the General Directorate of Public Health at the MOH which was the hub for the interaction of almost all workgroups involved in the policy. One channel was through allies in the public health network. Another channel was through their think tank, PWG. The third was through the Provincial Task Force on Prevention in Youth (PTPY) created by Quebec’s Premier. The fourth channel was through an inter-ministerial workgroup that the NPHD convened for the GAP formulation, and the fifth was the media. The use of any channel was not mutually exclusive, and some nutritionists made use of all five channels.

#### Develop evidence

Nutritionists worked to develop evidence on weight problems – defining them; and identifying their causal factors, their policy stakeholders, their roles and their priority interventions. Parameters and surveillance data were constantly monitored and evaluated^(40,41)^. They collaborated to review existing scientific evidence and to conduct original research. Developing evidence and framing occurred mostly when the PWG was active, between 2000 and 2006.

Nutritionists started with a review of the state of knowledge and a situation analysis to identify advocacy needs^(39)^. The review was utilised to raise awareness of the obesity epidemic from a public health perspective.^§7^ The authors called for the creation of an inter-sectoral group to work on a governmental plan on weight problems^(39)^. It laid the foundational stone for the PWG who worked on the dual mandate of developing a common vision and proposing an action plan to address weight problems^(41)^.

The PWG launched the Enabling Environments Project, a large project of review and consultations. Its aim was to identify the most promising and effective interventions for targeting environments to prevent and reduce weight problems^(40,46)^. The PWG likewise recommended a governmental action plan^(40)^.

Nutritionists also participated in a market analysis on weight loss products and services. The analysis revealed that < 1 % of inventoried services and products relied on a comprehensive approach of weight management and called for a prompt action^(47)^.

In parallel to the PWG work, the MOH consulted its public health network.^§8^ Workshops were organised by nutritionists and their co-workers to develop a consensus on effective interventions related to five intervention axes that included clinical preventive practices, food environments, physical activity environments, social norms and media. These were the precursors of the GAP intervention axes. Concurrently, nutritionists also participated in the identification of the intervention framework of the future governmental plan^(15)^. A nutritionist from the National Public Health Institute conducted a literature review on national obesity plans in similar countries^(48)^.

#### Communicate evidence

The knowledge gained helped nutritionists communicate their policy positions and advocacy materials to various audiences. Many of the PWG nutritionists had a marked contribution to the Annual Public Health Meetings.^§9^ Their first review^(39)^ was launched during the Annual Public Health Meetings and helped increase awareness and recruit allies.^§10^

The PWG communicated two main documents: *A Call for Mobilization* ^(41)^ and *A Call for Action* ^(40)^. The latter presented priority interventions and stakeholders’ responsibilities. It was launched at the Annual Public Health Meetings in the presence of senior public health authorities^§11^ and benefited from a large media coverage. The high visibility of weight problems attracted the media^(41)^. One nutritionist had a lot of media interventions^§13^ and was awarded the title *Scientist of the Year* in 2006 by local Canadian media for her contributions to understanding the policy issue as a public health problem^(49)^.

A *‘Guidebook on the principles of healthy weight management and on a critical analysis of weight-loss products and services’* was developed^(47)^ and endorsed by six professional regulatory bodies and one professional federation^(40)^. Other guides were added to the educational toolkit^(50,51)^ with the aim of regulating their market.^§12^

A major strategic communication was carried out by the ODNQ. Its president submitted a brief to the National Assembly of Quebec following the government consultation on healthcare access^(52)^. The ODNQ reaffirmed its commitment to prevention to ensure health system sustainability. The ODNQ brief echoed the recommendations of the PTPY and requested their immediate implementation^(52)^.

#### Participate in deliberative forums and negotiate an agreement

Nutritionists had a major role in the GAP negotiations. Having reached consensus on the internal framing of weight problems within their policy community, the challenge was to engage other coalitions. Their key ally, the NPHD, was the Assistant Deputy Minister for public health and had the ears of the health minister. He participated in most forums and helped them advance the policy issue. Four deliberative forums contributed significantly to the GAP (Fig. 2(d)).

##### Strategic planning workshop

This workshop was organised to plan future obesity policy after the National Program of Public Health 2003–2012 was adopted with few interventions to promote enabling environments^(53) §14^. Inspired by the successful public health advocacy leading to the enactment of Quebec Tobacco Act^(54)^, a consensus was established around a socio-political intervention known as the *Quebec Model of Obesity Prevention* ^§15^.

##### Ministry of Health and Social Services workshops

The five workshops held by the MOH with its public health network were the precursors of the GAP intervention axes and respected the legal public health framework in Quebec^(55)^. Nutritionists collaborated with their regional partners to review evidence, map the actual situation, identify potential partnerships and issue recommendations^§16,24^.

##### Provincial Task Force on Prevention in Youth

The change in the governing party following the elections in Quebec led to a series of events favouring the EEAC. The newly elected government^(56)^ held a provincial forum that revealed a favourable public opinion of prevention in children and youth^(56)^. A task force was created by the Premier in 2004 to advise the government on prevention^(57)^. The MOH was asked to support the PTPY and to recommend its membership. Nutritionists participated in the selection of PTPY members ensuring they shared similar views on the policy problem^§22^. The president of the ODNQ participated in the PTPY. Other nutritionists presented scientific evidence raising awareness of participants including ministerial delegates on the importance of enabling environments^§17^. The PTPY’s recommendations were aligned with most of the PWG’s recommendations including the development of a governmental plan.

##### Inter-ministerial workgroup

When the Premier commanded the plan, the NPHD convened ministerial delegates to discuss future interventions under each ministry’s mission.^§18^ The negotiation process was strenuous and challenging before a negotiated agreement was reached^§19^; some policy actors had divergent and sometimes opposing policy beliefs.^§20^ Some others were not mobilised around the policy issue.^§19^ The collaboration of policy actors from other coalitions was mitigated, and many were wary of the interference of health authorities in their ministerial missions.^§19^ Nutritionists channelled previous works to formulate the GAP.^§23,24^ They worked bilaterally with ministerial delegates and reviewed their ministries’ interventions in health promotion.^§21^ Negotiation strategies included sharing scientific evidence to influence delegates in their intervention choice, promoting inter-sectoral collaboration, using persuasive and emotive language and describing the situation as a public health crisis requiring urgent policy response as evidenced by the Premier’s request^(40)^.^§19,21,22^

### Power analysis

Distribution of power among coalitions favoured the nutritionists’ coalition. The EEAC’s control was threefold: their leadership in formulating the GAP, their control of the critical functions related to the development of health policies and the Public Health Act^(55)^, which entrusts the Minister of Health to advise other ministers on policies that promote health and well-being when deemed necessary^(55)^. The other coalitions struggled to maintain and safeguard their autonomy in a context where the EEAC was trying to reap the benefits of the plan. Having worked on the agenda setting and assumed the leadership of the formulation of the policy, the EEAC strongly supported the plan. Their goal was to maintain the health sector’s leadership in health promotion and disease prevention and to maximise the interventions of other coalitions under the GAP.^§22^ The GAP was an opportunity to introduce and oversee new interventions under other governmental missions. Therefore, the risk of having the EEAC gain control over other coalitions or impose its own interventions was increased and exaggerated by non-EEAC coalitions. Accordingly, their support to the plan was limited. Their goals were to protect their autonomy and to avoid the interference of the health sector in their ministerial missions.

The HLPAC’s goal was to preserve its leadership in promoting physically active lifestyles, to ensure the right balance between interventions on healthy diets and those on physically active lifestyles under the GAP and to maintain the lead of the school food policy.^§25^ The AFAC’s goal was to protect their autonomy and to safeguard the interests of the agri-food industry while maintaining a good level of collaboration with the health sector. Under the leadership of the Ministry of Agriculture, Fisheries and Food, the AFAC pre-emptively created a workgroup and developed their own ministerial action plan to propose under the GAP. This way, they achieved a dual objective: they increased the legitimacy of their proposed GAP interventions and they limited other coalitions’ interference. Other strategies conducted by the AFAC were to discredit and raise doubts on interventions proposed by the EEAC such as taxation of unhealthy foods. They only introduced voluntary interventions and rejected regulations targeting the agri-food industry.^§25^

The CDAC proposed a PPP to support GAP-related interventions at the community level^(57)^. They did not interfere in the formulation of the ministerial interventions under the GAP. Their goal was to have the plan adopted by the government enabling the establishment of a PPP that would allow them to operate the future healthy lifestyles fund^§25^.

### Influence on the agenda setting and formulation of the governmental action plan

Long-standing advocacy and commitment of engaged nutritionists led to their readiness to formulate the GAP. Multiple works to which they contributed converged in the GAP^§23,24^ (Fig. 2). Nutritionists built partnerships and created a cohesive policy community. Their think tank had been active for six years prior to the GAP adoption, and its advocacy was significant in influencing the agenda setting and policy formulation. Using scientific evidence and political strategies, they developed and communicated a public health perspective on the policy issue and drew political and public attention to it.

Nutritionists set the ground for the activities of the deliberative forums organised around the policy issue. Nutritionists negotiated and provided convincing scientific evidence to influence the beliefs and strategies of other advocacy coalitions, so that they formulated policy solutions that are aligned with the enabling environments paradigm.^§19,21^ A window of opportunity opened for their coalition with the change in government. The creation of the PTPY that recommended the governmental plan provided them with short-term opportunities. They controlled deliberative processes influencing the selection of participants. They integrated members with similar policy beliefs to the PTPY which provided them with a leverage over other coalitions in advancing their own policy solutions. They also influenced the members of the PTPY in understanding the problem and its causes, identifying stakeholders, defining the target population, setting priority interventions and acknowledging the need for policies that promote enabling environments^(57)§17^.

The GAP integrated the nutritionists’ core beliefs (Table 3). However, only those regulatory interventions for which the MOH bears responsibility, such as regulating the weight-loss industry, were introduced under GAP. The plan fell short of introducing regulatory actions under other ministries’ missions, for instance, those targeting the agri-food industry to improve food environments. Rather, incentives were proposed to increase awareness and voluntarily improve the industry’s practices. Nutritionists and their colleagues made sure to extend the GAP target population to include young adults and their families hoping to reach the population at large.^§26^ The GAP was a huge government workshop for an unprecedented inter-sectoral collaboration promoting a whole-of-government approach to obesity prevention in a learning context that challenged the existing silos^§27(58)^.

## Discussion

When practising in public health, nutritionists participate in public policymaking^(1,5,9)^. Effective advocacy increases one’s power through an enhanced control over resources which increases the influence on the policy process^(1)^. It allows nutritionists to influence policymaking and to integrate evidence, their values and interests in public policies. This is particularly important in a context where the food industry advocacy capacity significantly outweighs theirs^(13,59)^.

This qualitative study improves the understanding of the political and policy processes around the agenda setting and formulation of a governmental obesity policy which can benefit public health advocates and help them improve their mobilisation efforts with the government^(60,61)^. The study analyses the role of nutritionists in the agenda setting and the formulation of the GAP using a conceptual framework drawn from political science. Frameworks provide categories of analysis of the drivers enabling or constraining the emergence of issues on the policy agenda^(60)^. A strong theoretical grounding is likely to promote explanatory power and transferability of a study to another context^(21,62)^. In single-case studies, using theory enhances external and construct validity^(38)^.

Although the predominant use of a single framework has been reported in research on agenda setting in public health^(60,62,63)^, scholars have recently argued that the combination of complementary frameworks provides a comprehensive understanding of agenda setting drivers^(60)^. Each framework focuses on a different set of assumptions on the key drivers for policy change. Therefore, combining frameworks can best illuminate the complexities characterising social problems and policies^(60)^. However, this comes with methodological challenges and complexities^(64)^.

A combination of complementary policy frameworks helped explain the agenda setting or the policy change of several policies in various high-income contexts^(21,60,62,65,66)^. In this case study, the use of two theoretical perspectives and concepts helped generate a comprehensive understanding of the agenda setting and the formulation of the GAP. The ACF analysis helped understand the nutritionists’ policy beliefs and their strategies based on those beliefs. The political analysis helped to analyse the political goals of nutritionists as one driver of their strategic behaviour in a power distribution where they were dominant. The use of a strong framework facilitated data coding and interpretation^(67)^, while coding for emergent themes decreased the overreliance on theory and provided a balance between inductive and deductive codes^(67)^.

The current study shows that nutritionists influenced the technical and scientific aspects of decision making. They built a shared understanding on an internal policy frame. Internally, obesity was portrayed as a problem caused by obesogenic environments jeopardising population health and public finances. An alignment around a common internal frame drives the political commitment to nutrition^(63)^. Nutritionists communicated evidence with a sense of crisis, highlighted current policy failures and garnered support from actors outside the direct scope of the problem. This is a crucial precursor for policy change^(37)^.

Externally, nutritionists opposed the frames of other coalitions who believed that interventions should target children as a priority. This framing resonated less with their external audiences including policymakers. By using compelling issue frames that resonate well with policymakers and aligning solutions with their beliefs and the government’s objectives, advocates can influence the political aspect of decision making^(21)^. Frames that resonate successfully with policymakers include safeguarding children’s health, communicating a sense of crisis^(1)^ and providing the population with informed choice^(21)^. In fact, most national nutrition policies in high-income countries target children^(19)^. Externally, ministers were less familiar with the importance of environments in shaping individual choices when the GAP was developed^(68)^. Advocacy efforts of nutritionists were less oriented towards senior policymakers, which has also been reported in the Australian context^(59)^.

Nutritionists emphasised evidence as the gold standard to advance their policy proposals. The extent to which evidence is available, communicated and accepted is a driver for political commitment for nutrition^(63)^. Higher levels of evidence are required when issues are strongly contested, such as food regulations to prevent obesity^(63)^. Nutritionists used scientific evidence to promote their policy position while debating and negotiating with other coalitions, particularly in the inter-ministerial workgroup. While various studies and reviews show that evidence has been used by advocacy coalitions to support their policy position^(8,69)^, other studies have found that the use of evidence alone as an advocacy strategy had a limited influence on policymaking^(20,22)^.

Nutritionists developed strategic partnerships that are a key factor in influencing the policy process^(20,22)^. However, the high cohesion that characterised their policy community was faced with a fragmented collaboration with their ministerial counterparts. Food- and nutrition-related policies need a wide array of actors^(1,14)^. Based on our findings, we recommend improving the input legitimacy of deliberative processes organised around obesity policy. The input legitimacy refers to transparency, inclusive deliberation and fair process^(70)^. Integrating actors from other policy subsystems to deliberative processes can foster coherence, inter-sectoral collaboration and innovative solutions. This was the case with taxing sugar-sweetened beverages in Mexico^(14)^ and restricting fatty meats in Ghana^(71)^ where the Finance and Trade ministries were key collaborators, respectively. An overarching component of food and diet policies, according to various scholars, professional bodies, national food policies and dietary guidelines, is sustainability^(3,14,19,21,72–81)^. For effective advocacy, nutritionists should go beyond traditional silos and extend their partnerships to strategic stakeholders across diverse policy subsystems.

Our study had some limitations. Recall bias might have occurred as participants were reporting on past events, beliefs and strategies. To overcome recall bias, we provided them when needed with support documents, such as copies of the plan, ministerial reports and lists of participants of forums. Although the number of interviewed nutritionists is modest, we are confident that their extensive implications and affiliations allowed us to capture all of the major nutritionists’ advocacy practices that contributed to the GAP.

## Conclusion

The current study is the first research to analyse the contribution of nutritionists as policy actors advocating for a governmental plan for the promotion of healthy lifestyles and prevention of weight problems from both an advocacy coalition and a political analysis perspective. The empirical insights gained from the current study help build the capacity of the nutrition and public health work force and foster more evidence-based advocacy. The importance of this research is in its capacity to inform the body of knowledge on nutritionists’ advocacy and to reinforce their current and future advocacy understanding and practices. We also hope it will interest scholars to strengthen their research in nutrition advocacy, going beyond understanding practices to monitoring and evaluating them.

## Supplementary material

For supplementary material accompanying this paper visit https://doi.org/10.1017/S1368980021004997. click here to view supplementary material
