# Supplementary material for: Nutritionists as policy advocates: the case of obesity prevention in Quebec, Canada
Source: Public Health Nutr. 2021 Dec 27;25(7):2011–24. doi: 10.1017/S1368980021004997 (PMC9991765; doi:10.1017/S1368980021004997)
Supplement: Supplementary file 1 [file S1368980021004997sup.zip › S1368980021004997sup001.docx]

**Table 1. Detailed Interview Guide (Grille d’entrevue détaillée)**

**Date : _________________________________ Ministère(s)/ autres organisations : ________________________**

**Sexe : F H Poste(s) occupé(s) : _____________________________________**

**Profession : _____________________________ Année(s) d’expérience : __________________________________**

| **Thèmes ACF** | **Questions** | **Sous-questions** | **Commentaires** |
| --- | --- | --- | --- |
| **Sous-système des politiques de prévention de l’obésité (SSPPO)/ *coalitions plaidantes : acteurs*** | Quelles sont les **personnes impliquées** dans les politiques de prévention de l’obésité (problèmes reliés au poids PRP, promotion d’une saine alimentation SA et d’un mode de vie physiquement actif MVPA) au Québec ? | 1. Pouvez-vous me décrire **votre implication** dans le dossier de la prévention de l’obésité : prévention des PRP, promotion d’une SA et d’un MVPA ?   **Probe :** emploi actuel, emplois antérieurs, organisation et secteur d’emploi actuels, organisations et secteurs de travail antérieurs, implication locale, régionale, nationale, canadienne ou internationale, évolution de carrière depuis 1996   1. Qui sont les **personnes et quelles sont les organisations avec qui/avec lesquelles vous avez collaboré** sur la thématique de la promotion d’une saine alimentation et de l’activité physique depuis 1996 ?   **Probe :** personnes et institutions/organisations avec une collaboration actuelle et antérieure | Les informations sur l’implication de l’informateur clé aident à :   - Le situer dans un **groupe d’acteurs selon sa profession et son organisation**. - Connaitre les acteurs, professions et les organisations impliquées dans le SSPPO au Québec - Reconnaitre les acteurs et les organisations matures dans le sous-système |
| **Coalitions plaidantes / *croyances*** | Quelle est votre perception de la problématique de l’obésité ? | 1. Parlez-moi de votre **perception de la problématique de l’obésité**.   **Probe :** Ampleur et gravité, aspects les plus importants de la problématique, problème à l’échelle de la société ou à l’échelle de l’individu   - 1. Comment votre perception a évolué **à travers le temps** ?   2. Pourquoi ?  1. Quelles sont selon vous les **causes** de l’obésité ?   **Probe :** choix personnels, causes physiologiques et génétiques, environnement obèsogène   1. A quel point pensez-vous qu’il **soit possible de réduire les taux d’obésité** ? | Les informations sur les croyances de l’informateur clé aident à :   - Comprendre sa perspective sur la problématique de l’obésité - Identifier la stabilité ou le **changement** de ses croyances et l’expliquer - Comprendre le point de vue de l’informateur clé sur la **capacité de la société** de résoudre la problématique |
| **Coalitions plaidantes / *croyances***  ***(suite)*** | Quelles sont vos préférences vis-à-vis les politiques de prévention de l’obésité ? | Quelle est votre **perception des solutions** à la problématique de l’obésité qu’il faut privilégier ? Quelles sont vos **préférences** vis-vis les politiques de prévention des PRP et de la promotion d’une SA et d’un MDVPA ?  **Probe :** promouvoir la saine alimentation et l’activité physique au niveau des individus/ au niveau des collectivités / autres ; améliorer les environnements (physique, socioculturel, politique, économique) ; améliorer les services aux personnes prises par la problématique du poids.   - 1. Comment votre perception sur les solutions de la problématique de l’obésité a évolué **à** **travers le temps**   **Probe :** politiques à l’échelle de l’individu (améliorer le comportement, les services pour les personnes prises avec le problème du poids) vs politiques à l’échelle sociétale (améliorer les environnements)   - 1. Pourquoi ?   **Probe :** information scientifique, l’exemple d’autres pays, autres   1. **Avec qui vous vous entendez** sur les solutions de l’obésité et avec qui vous avez des différents ? Comment ceci peut-il affecter l’élaboration et l’adoption des politiques de prévention de l’obésité ? 2. Quels sont les **instruments de politique** que vous privilégiez ou que vous trouvez prioritaires :   **Probe :** lois, règlements, incitatifs financiers, taxation, crédits d’impôts, éducation, autres | Ces informations aident à :   - Situer l’informateur clé dans une **coalition d’acteurs** selon ses croyances de politiques. - Connaitre les « **goals** » des acteurs - Comprendre leurs préférences vis-à-vis les politiques à privilégier comme solution à l’obésité (qui représentent la « colle » de la coalition) - Comprendre si leurs croyances de politiques ont changé à travers le temps (**APPRENTISSAGE**) et le cas échéant les raisons du changement ; questionner le ROLE DE **L’INFORMATION SCIENTIFIQUE** - Connaitre les instruments de politique à privilégier - Connaître les **alliés et les opposants** de l’informateur clé |
| **Coalitions plaidantes / *croyances***  **(suite)** | Quel est le rôle que devra jouer le gouvernement et les divers secteurs de la société dans les solutions proposées et particulièrement votre secteur ? Quel est votre perception de la coordination de cet effort ? | 1. Selon vous **qui peut rendre ces solutions possibles** ? Pourquoi ?   **Probe :** perception du rôle du gouvernement, des institutions, société civile, groupes de pression, divers secteurs de la société (privé, public, associatif etc.) ; partage des rôles et des responsabilités   - 1. Que pensez-vous du rôle du gouvernement ? Quels sont les ministères concernés et à quel niveau de l’état ? Quel est le rôle des élus ?   2. Que pensez-vous du rôle du secteur privé ? de l’industrie ?   3. Que pensez-vous du rôle d’autres secteurs (associatifs, ordres professionnels, société civile, groupes d’intérêt, secteur académique, experts, média etc.)  1. Que pensez-vous de l’importance de la coordination des actions de ces secteurs ? 2. Comment pensez-vous que « votre ministère / organisme » doit être impliqué dans la prévention des PRP, la promotion d’une SA et d’un MDVPA ? pourquoi pensez-vous que c’est le rôle qui doit lui être attribué ? | Ces informations aident à :   - Comprendre la perception de l’informateur clé du partage de l’autorité entre les différents niveaux et secteurs du gouvernement - Comprendre la perception de l’informateur clé du partage de l’autorité entre les différents secteurs de la société - Comprendre la perception de l’informateur clé sur l’importance de la coordination des actions/acteurs - Comprendre la perception de l’informateur clé sur le rôle de son ministère / organisation |
| **Coalitions plaidantes / *croyances***  **(suite)** | Quel(s) est/ sont les groupes à prioriser dans les solutions propo-sées ? | 1. Est-ce que selon vous les actions visant à la prévention des PRP, la promotion d’une SA et d’un MDVPA doivent viser un groupe en particulier ?   **Probe :** jeunes, groupes vulnérables etc. | Permet d’identifier les groupes ou l’action est prioritaire selon l’informateur clé |
| **Sous-système des politiques de prévention de l’obésité / *coalitions plaidantes : acteurs*** | Qui sont les acteurs clés du PAG et quelles sont leurs préférences de politiques ? | 1. **Qui** a déclenché l’intérêt dans un plan d’action gouvernemental ? Qui partageait l’intérêt pour un plan d’action gouvernemental ?   **Probe :** individus attachés à différents secteurs et ministères (gestionnaires, professionnels de la santé publique etc..), premier ministre, ministres et sous-ministres, média, société civile (opinion publique), chercheurs, organismes communautaires, groupes d’intérêt, autres.   1. Pouvez-vous me nommer les personnes qui selon vous avaient eu le plus d’influence dans le cheminement du PAG ?   **Probe :** ministres, sous-ministres, premier ministre, employés ministériels, membres d’organismes communautaires ou provinciaux, de groupes d’intérêt, médias etc.   - 1. Pourquoi selon vous ces personnes avaient cette influence-là ?   **Probe :** position de pouvoir ou d’autorité, leadership, ressources monétaires, popularité, crédibilité, connaissances, autres.   1. Quelles politiques ces personnes-là essayaient-elles d’avancer ? | - Permet d’identifier les champions du PAG et les acteurs dominants du PAG. (Plus impliqués et / ou avec plus de pouvoir). A RECHERCHER dans tout le cheminement du PAG : du **plaidoyer pour le PAG, à l’élaboration, à l’adoption** du PAG - Permet d’identifier les « goals » des acteurs dominants / champions du PAG |
| **Sous-système des politiques de prévention de l’obésité / *coalitions plaidantes : acteurs et coordination* *des acteurs*** | Quel est votre implication initiale au PAG et comment avez-vous assuré une coordination avec les autres acteurs ? | 1. Comment et pourquoi (dans quelle capacité) a-t-on fait appel à vous pour contribuer au PAG ?   **Probe :** votre poste à ce moment ; l’organisme auquel vous appartenez ; et qui vous a contacté   1. Qui sont les personnes, les organismes et les ministères avec qui vous avez collaboré pour le PAG ?   **Probe :** équipe de travail (composition de l’équipe, membres de l’équipe), collaborateurs dans le même département, collaborateurs dans d’autres départements de la même organisation, collaborateurs dans d’autres organisations (public, privé, associatif), collaborateurs dans d’autres secteurs, partenariat, comité, tables nationales, autres.   1. Comment étaient-ils engagés dans le processus du PAG ?   **Probe :** Engagement dans le plaidoyer, élaboration, rédaction, révision, adoption, évaluation, coordination, répondant ministériel, consultant, expert, autres. | Les informations sur le mode d’implication de l’informateur clé dans le PAG et de son équipe de travail aident à :   - Reconnaitre l’acteur/le groupe d’acteurs qui l’a engagé dans le PAG - Le situer dans une coalition d’acteurs selon sa profession et son organisation - Reconnaitre les collaborateurs et les alliés de l’informateur clé - Reconnaitre les éléments et les structures de coordination |
| **Sous-système des politiques de prévention de l’obésité / …**  **(suite)** | Comment se déroule la coordination entre vous et les autres acteurs ? | 1. Comment pouvez-vous décrire votre relation avec vos collaborateurs / alliés ?   **Probe :** relations INTERNES et EXTERNES (à l’interne dans le ministère/organisme ET à l’externe)   1. Comment votre collaboration a évolué à travers le temps ?   **Probe :** éléments favorisant la collaboration , obstacles, barrières et conflits, défis à relever, changement des éléments et des structures de coordination, de l’engagement des individus à travers le temps, autres. | Permet de chercher une information plus détaillée sur le niveau de coordination / conflit entre les acteurs, sur les règles et les structures de coordination, ainsi que l’évolution de la coordination et la volonté des acteurs à s’engager |
| **Sous-système des politiques de prévention de l’obésité / *coalitions plaidantes : ressources*** | Qui a fourni les ressources financières pour permettre votre implication au PAG ? | 1. Qui a financé votre travail sur le PAG ? Quels sont les coûts associés à ce travail ?   **Probe :** l’organisme là où l’informateur clé travaillait, un financement ponctuel, autres. | Permet d’identifier les sources des ressources financières engagées dans l’embauche de l’informateur clé à travailler sur le PAG |
| **Sous-système des politiques de prévention de l’obésité / … *(suite)***  **Sous-système des politiques de prévention de l’obésité / …**  **(suite)** | Quelles sont les ressources dont vous disposiez ? Quelles sont les ressources dont disposaient les acteurs du PAG (alliés et opposants) ? | 1. De quel type de soutien vous avez bénéficiez pour faire avancer le PAG ? Quelles sont les ressources qui étaient à votre disposition ou que vous avez engagées pour vous soutenir dans votre implication dans le PAG ? Comment ces ressources ont-elles été utilisées et coordonnées, voire non utilisées ?   **Probe :** soutien politique - personne ayant une autorité formelle juridique (législateur, responsable de l'agence, etc.), ressources financières (allocations spécifiques), experts, leadership, opinion publique (sondages d'opinion), troupes mobilisables (partisans, adversaires), informations, médias ; utilisation des ressources, coordination des ressources (centrale versus régionale ; sectorielle versus gouvernementale)   1. Quelles sont les ressources dont disposaient vos partenaires et alliés (ou qu’ils ont engagées) ? Comment ces ressources ont-elles été utilisées et coordonnées, voire non utilisées ?   **Probe :** les ressources financières (allocations spécifiques), experts, leadership, opinion publique (sondages d'opinion), troupes mobilisables (partisans, adversaires), personne ayant une autorité formelle juridique (législateur, responsable de l'agence, etc.), connaissance, médias, utilisation des ressources, coordination des ressources (centrale versus régionale ; sectorielle versus gouvernementale)   1. Quelles sont les ressources dont disposaient vos opposants (ou qu’ils ont engagées) ? Comment ces ressources ont-elles été utilisées et coordonnées, voire non utilisées ?   **Probe :** les ressources financières (allocations spécifiques), experts, leadership, opinion publique (sondages d'opinion), troupes mobilisables (partisans, adversaires), personne ayant une autorité formelle juridique (législateur, responsable de l'agence, etc.), informations, médias ; utilisation des ressources, coordination des ressources (centrale versus régionale ; sectorielle versus gouvernementale) | Les informations sur les ressources des acteurs aident à connaitre :   - Les ressources mises à la disposition ou développées ou utilisées pour le PAG - Comment ces ressources ont été engagées ou utilisées (STRATEGIES D’UTILISATION DES RESSOURCES) - Quelle coalition en bénéficiait le plus (POUVOIR) - Les CONTRAINTES et les OPPORTUNITES reliées aux ressources |
| **Evènements externes & évènements internes / contraintes et ressources** | Y a-t-il eu un évènement qui a déclenché l’intérêt dans le PAG ? Autres facteurs qui ont favorisé, facilité ou qui ont contraint le PAG ? | 1. Selon vous **qu’est-ce qui** a déclenché l’intérêt dans un plan d’action gouvernemental pour la promotion des saines habitudes de vie ?   **Probe :** Taux d’obésité alarmant, préoccupation des gens, influence d’autres programmes ou politiques (provinciales, nationales ou internationales) santé des jeunes, médias, opinion publique, pression des groupes d’intérêt, d’organismes communautaires, préoccupation mondiale et nationale, événement spécifique au niveau international, national ou provincial etc.   1. Quels sont les facteurs qui ont facilité le PAG ? Pouvez-vous me décrire comment ?   **Probe :** contribué positivement au processus politique (plaidoyer, élaboration ou adoption du PAG) - changement au sein du parti au pouvoir, de l'opinion publique, de la technologie, d'autres sous-systèmes, influence d’autres programmes et politiques, autres   1. Quels sont les facteurs qui ont entravé le PAG ?   **Probe :** contribué négativement au processus politique (plaidoyer, élaboration ou adoption du PAG) - changement au sein du parti au pouvoir, de l'opinion publique, de la technologie, d'autres sous-systèmes, influence d’autres programmes et politiques, autres | Ces informations aident à connaitre les événements INTERNES ou EXTERNES qui ont influencé le PAG dans toutes ses étapes (SE CONCENTRER SUR LE PLAIDOYER, ELABORATION & ADOPTION) et à comprendre comment ces événements ont pu créer des contraintes ou des opportunités aux acteurs |

| **Sous-système des politiques de prévention de l’obésité / *coalitions plaidantes : stratégies*** | Comment avez-vous contribué au processus politique du plaidoyer, de l’élaboration, et de l’adoption du PAG et quels étaient les défis que vous aviez dû relever ? Qu’est-ce que vous auriez dû faire mieux ? | 1. Pouvez-vous me décrire votre rôle ou votre implication dans le plan d’action gouvernemental 2006-2012 pour la promotion des saines habitudes de vie ? Comment pensez-vous avoir influencé le PAG ?   **Probe :** plaidoyer, élaboration, adoption ou prise de décision, décideur, autres.   - 1. Quelles sont les actions auxquelles vous avez contribuées ou que vous avez entamées qui ont fait avancer le PAG ?   2. Quelles sont les actions auxquelles vous avez contribuées qui ont entravé le progrès du PAG ?   3. Quelles sont les actions que vous auriez dû faire mais n’aviez pas faites ?   4. Qu’est-ce qui vous a empêché de les faire ?   5. Pouvez-vous me décrire les actions qui ont été menées ou que vous avez menées dans le cadre du PAG pour prendre en considération les groupes à cibler en priorité dans la problématique de l’obésité ?   **Probe :** plaidoyer ou élaboration de dispositions dans les politiques qui tiennent compte de ces personnes etc.   1. Quels sont les arguments que vous avez utilisés pour faire avancer le PAG ? 2. Quelles sont les informations que vous avez utilisées pendant votre implication dans le PAG et quelles étaient leurs sources ?   **Probe :** littérature scientifique, autres plans d’action nationaux, experts, opinion publique, données sur le Québec, INSPQ, Statistiques Canada, ISQ, autres   1. Quels étaient les difficultés, obstacles ou les défis majeurs que vous avez eus pendant l’élaboration du PAG ? pendant l’adoption du PAG ? Comment avez-vous fait face à/surmonter ces défis ?   **Probe :** manque de ressources, changements de priorité, manque de coordination, changement de leadership, autres.   1. Si vous souhaitez pouvoir influencer le PAG différemment comment l’auriez-vous fait et qu’est-ce que vous auriez changé ? | Ces informations permettent de rechercher et comprendre :   - Les stratégies mises en œuvre par l’informateur clé pour le plaidoyer, l’élaboration et l’adoption du PAG (stratégies intentionnées, émergentes, réalisées, non réalisées, délibérées ; Stratégies gagnantes & perdantes) ; - Les CONTRAINTES et les OPPORTUNITES qui ont favorisé ou contraint l’action de l’informateur clé - Les arguments utilisés par l’informateur clé pour appuyer ses propos - Les sources d’information utilisées par l’informateur clé - Comment il/elle aurait pu faire mieux |
| --- | --- | --- | --- |
| **Sous-système des politiques de prévention de l’obésité / *coalitions plaidantes : stratégies***  ***(suite)*** | Comment est-ce que vos collaborateurs ont-ils contribué au processus politique du plaidoyer, de l’élaboration, et de l’adoption du PAG et quels étaient les défis qu’ils avaient dû relever ? Qu’est-ce qu’ils auraient dû faire mieux ? | 1. Pouvez-vous me parler des actions auxquelles vos alliés ou collaborateurs ont contribuées ou qu’ils ont entamées qui ont contribué au PAG plus que d’autres ? qui ont entravé le progrès du PAG ? si oui lesquelles ? Quelles sont les actions qui, selon vous, vos alliés ou collaborateurs auraient dû faire mais n’avaient pas fait et qu’est-ce qui selon vous leur a empêché de le faire ? Quelle est votre perception de leurs actions ?   **Probe :** plaidoyer, élaboration, adoption ou prise de décision, décideur, autres. | Ces informations permettent de rechercher et comprendre :   - Les stratégies mises en œuvre par les alliés/collaborateurs de l’informateur clé (stratégies intentionnées, émergentes, réalisées, non réalisées, délibérées ; Stratégies gagnantes & perdantes) ;   Les CONTRAINTES et les OPPORTUNITES qui ont favorisé ou contraint les actions de ses alliés/ collaborateurs selon l’informateur clé |
|  | Comment est-ce que vos opposants ont agi et comment avez-vous réagi en réponse ? | 1. Quelles sont les stratégies que vos opposants ont utilisées pour influencer le plan d’action gouvernemental à travers le temps ?   **Probe :** réduire les ressources : financières, effectifs en ressources humaines, autres.   1. Quelles étaient vos réactions à leurs stratégies ? | Ces informations permettent de rechercher et de comprendre les stratégies mises en œuvre par les opposants de l’informateur clé ainsi que sa réaction à leurs stratégies |
| **Sous-système des politiques de prévention de l’obésité / *Règles institutionnelles*** | Quels sont les changements institutionnels que le PAG a favorisés ou empêchés ? | 1. Quel changement le PAG (l’adoption du PAG) a-t-il favorisé au niveau des institutions ? Quel changement le PAG a empêché au niveau des institutions ? Que pensez-vous de cet effet ? | Cette question permet de connaitre la perception de l’informateur clé des changements institutionnels favorisés/empêchés par le PAG et sa perception de ces changements |
| **Sous-système des politiques de prévention de l’obésité / *Impact de la politique*** | En suivant votre définition du succès du PAG, est-ce que le PAG a réussi selon vous ? | 1. Quelle est votre définition du succès du PAG et est-ce que le PAG a réussi selon vous ? 2. Quelles sont les conséquences non voulues du PAG selon vous ? | Cette question permet de rechercher la perception de l’informateur clé sur   - Le succès du PAG   Ce que le PAG a engendré comme effets non désirés |
| **Sous-système des politiques de prévention de l’obésité / *Impact du sous-système (sur les évènements externes, contraintes et ressources des acteurs)*** | Quelles leçons l’expérience du PAG vous a-t-elle appris (e)? | 1. Quelles sont les leçons que vous avez apprises à travers le processus du PAG ? 2. Comment est-ce que le PAG peut affecter les politiques futures en prévention de l’obésité (PRP, promotion d’une SA et d’un MVPA) ? | Cette question permet de rechercher et de comprendre la perception de l’informateur clé sur le processus politique global du PAG ; sa perception sur l’effet de ce processus sur les politiques futures |
