# Supplementary material for: Nutritionists as policy advocates: the case of obesity prevention in Quebec, Canada
Source: Public Health Nutr. 2021 Dec 27;25(7):2011–24. doi: 10.1017/S1368980021004997 (PMC9991765; doi:10.1017/S1368980021004997)
Supplement: Supplementary file 1 [file S1368980021004997sup.zip › S1368980021004997sup002.docx]

**Table 2.** Illustrative quotes of Key Informants

| Theme | Subtheme | Illustrative Quote^§^ |
| --- | --- | --- |
| Policy core beliefs of coalitions | Beliefs regarding the causes of obesity and/or policy preferences to address obesity (1) | *“Obesity is an environmental problem […] it is not up to the individual to change environments it is the role of public policies.”*  *Government consultant (2016) - EEAC member* |
|  | Beliefs on causes of obesity and/or policy preferences to address obesity (2) | *“Doing physical activity or paying attention to your diet are individual choices [...]. The ultimate choice, it is the individual who makes it […]. An example that I will give you, at the level of the Ministry of Health, they believed a lot in the positive effect of a tax on junk food and I told them that I did not think that it was an effective intervention when it comes to food, especially that taxing food that is perceived to be less healthy can have a negative impact on the less well-off. The thing to remember is that, unlike smoking, eating is not a choice, and that is what I told them at the time. So, I repeat it to you: eating is not a choice and there is also a limit to what people can pay. So, I think it is necessary, and I do still think it is, I thought that it was necessary to intervene more on raising awareness and informing people”.*  *Government director (2016) – AFAC member* |
|  | Beliefs on causes of obesity and/or policy preferences to address obesity (3) | *“It is clear that physical activity is not promoted solely for the purpose of losing weight or for tackling the problem of obesity […]. If people are physically active even if they have excess weight, it is not so serious. It is not a problem by itself if people are active and have a healthy diet [...]. According to the physical activity promotion network, to see it, to see the link obesity-physical activity, it is clear that initially, it is not that there was divergence, but the link was not automatic […]”.*  *Government professional (2016) – HLPAC member* |
|  | Beliefs on causes of obesity and/or policy preferences to address obesity (4) | *“What happens in Maria in Gaspésie is not at all the same reality as Verdun here in Montréal; and the principle of communityship, it is the offer that is important, and we did not want to come up with, well, ‘you should work on food deserts’ when it was not at all their problem”.*  *Anonymous (2016) – CDAC member* |
| Build & reinforce partnerships | Sharing the same beliefs on the policy problem (5) | *“…There has been a journey through the PWG, it still lasted several years […], then, in the end, there was a real cohesion […] the first condition […] was first to share this vision very strongly inside the group and then throughout the years it happened.”*  *Government consultant (2016)* |
|  | Recruiting a key ally, the NPHD (6) | *“There were a lot of people working [on the obesity epidemic], in fact almost all […] were nutritionists and they had more or less recruited me given my experience in the tobacco case to see how we can put this problem in the public place […]. And, so with the lobbying experience […] on the tobacco case, I had from the start worked with the PWG to help them make it a file that has its place in public policy […].*  *NPHD (2016)* |
| Problem characteristic | The need to raise awareness on a public health perspective on obesity in Québec (7) | “[..] At that time here in Québec and almost everywhere there was no public health perspective on obesity, there was none because it was a problem that can be described as orphan, meaning that in public health we do not care about it. We care about tobacco, we deal with road accidents, we take care of anything, HIV, but we do not care about obesity as a public health problem. Obesity is a clinical problem that physicians are interested in, dietitians or kinesiologists […].  Government Consultant (2016) |
| Conduct research and develop evidence | The MOH consultation/ workshops with its public health network (8) | *“When Mayor Perrault [in reference to the President of the PTPY] was doing his works, [the NPHD] said […] ‘there is some work being done within the civil society, something will definitely come out of the recommendations’. He convened […] people from the Institute, people from the MOH, people from the network […] and then he asked: ‘how can we start working to prevent obesity? it is urgent, we must work on diet, we must work on the physically active lifestyle, body image is important, how can the public health network contribute in no time?’ we have held workshops […]”*  *Government professional (2016)* |
| Communicate policy positions and advocacy materials | Annual participation in the APHM (9) | *“We never missed training days at the Annual Public Health Meetings, because […] it was necessary that all public health actors understand precisely, to create this movement […] everyone in their region must be convinced, master this new paradigm to be able later to disseminate and convince decision-makers and actors in their region. So, we used the Annual Public Health Meetings to do this and then, from year to year, we progressed [...]”*  *Government consultant (2016)* |
|  | Communicate the first review and engage stakeholders (10) | *“… We wrote it [the document] to sensitize public health decision-makers we know, to start with, to have friends with us, to be able to move forward and at the time, I did not know it but I was in […] the agenda setting […] first, it takes a reading committee […]; on the reading committee you choose the people, very important, the list of people who will read the document, and these people, you are likely to convince them of something, sensitize them and then allow them to be open to new problems […]”*  *Government Consultant (2016)* |
|  | Communication of advocacy documents to key stakeholders and to the media (11) | *“… In fact, the last one [PWG document] was launched during the Annual Public Health Meetings in the presence of […] the assistant deputy minister at the time and several directors of public health as well; we saw at that time that we started to have a lot of interest; there was a nice press coverage, then there was beyond the press coverage, the interest of the public health directorates, public health directors also […]”*  *Government professional (2016)* |
|  | Development & communication of a guidebook on weight loss products and services (12) | *“So, we had a project on the weight loss products and services […]. Ultimately our intention was to try to get a regulation on this. […] There is very little experience in the whole world in regulating weight loss products and services. […] It is very difficult, the lobby of private companies, and very powerful. […] We could not present the project with this agenda because it had to be educational. […] Our intention was […] to do the said educational program, but to allow us to document, then after that, to try to find the means to bring in lawyers, people […] with legal training in drafting bills […]. So, in the meantime, we developed guides for doctors […] a guide for women, the public […] We did a lot of very interesting things and of a very good quality.”*  *Government consultant (2016)* |
|  | Contribution of one dietitian to the media (13) | *“How many hours I spent talking to reporters to help them prepare their research papers; I spent hours and hours […]. I was very available, everyone gave my name […] and well it works, because you have served them well once, so they call you next time. I have done a lot! [in reference to media interviews]”.*  *Government consultant (2016)* |
| Participate in deliberative forums and negotiate agreements | The need to conduct a strategic workshop on obesity policy (14) | *“Then in 2002, […] the first National Public Health Program, […] which must exist by virtue of […] the Law of Public Health adopted in 2001[…], arrives. It was a little discouraging to see that there is not much […] on weight problems, perhaps for the reasons I have mentioned here: it was the lack of evidence on the effectiveness of interventions.”*  *NPHD (2016)* |
|  | Strategic planning workshop and Learning from tobacco advocacy & experience (15) | *“We, the PWG, […] we want to act concretely in the prevention of obesity […]; it is not a round table, it is a think tank and it wants to lead to action. So, at some point, we did a strategic planning workshop […]. We invited […] our political strategist […]. And there, […] he gave us a drawing of how to get organized, based on the tobacco model and what we called the Québec model […] and […] he said […] we will take the example on tobacco: it takes a socio-political coalition, […] its job is to claim public policies […]to react to the media, to go to the ministers' offices, similarly to the tobacco coalition. It takes an organization that is softer like the ‘Québec council on tobacco and health’, the ‘Québec Council on weight and health’, […], therefore, an organization that will have the mission to promote the vision of enabling environments […]. After that, well, it takes a team of expertise […]. All this will conquer to get it [in reference to a governmental action plan]”.*  *Government consultant (2016)* |
|  | The MOH workshops with its public health network (16) | *“We had five projects with the network. […] It was a large mobilization. We asked all the regions for contribution […]. We had an axis that was on diet, a focus on the physically active lifestyle […], we had a focus on social norms and communications […]; in fact, it is special because the workshops’ titles [..] have almost given rise to the GAP axes; in fact, the work was to say ‘what are the solutions to put forward in the five areas?’”*  *Government professional (2016)* |
|  | Communicating evidence on obesity during the meetings of the PTPY (17) | *“[…] so, we will present […] the famous presentation on the prevalence of obesity, then we are collectively gaining weight with the slides of the United States, then people get shocked […].’How to explain that? It's not the individuals, it's the environment that is changing’. Then […] you show them […] the reduction in energy expenditure, the transformed food, […] then I obviously add all the issues of body image, always […]! And then […] ‘what should we do? Well, everyone must do something: a school director, a mayor, a company manager etc.’ Then, well, there is something for everyone. We basically presented the outline of a collective action plan”.*  *Government consultant (2016)* |
|  | Creation of an inter-ministerial work group, a key strategy of the NPHD (18) | *“The Minister of Health at the time, receives the recommendations of this external group [in reference to the PTPY], he tells me […] ‘I want an action plan on these recommendations’ […] “I want a plan of action within three months” […]. We were already 5-6 ministries because […] I had started convening them before; when the GAP arrived, it gave me a good reason to meet, we tried to influence them, ‘what are we doing already? Then, what could we do?’ So, we revitalized our committee […].”*  *NPHD (2016)* |
|  | Negotiations between delegates to the inter-ministerial work group (19) | *“It's difficult because normally you do not start from previous works so quickly to conclude in six months. You give time to know each other […] when I said earlier there were some ministries that were involved to support the Perrault work group [in reference to the PTPY], these people were on our working group also for the GAP elaboration. So, they were aware, […], of the problem, had attended as an observer at the Perrault report level. So, when they found themselves in inter-ministerial discussions, they were more prepared; others were not. […]. So, for them it was all new. therefore, it has been a lot of work bilaterally with the ministries; uh, a lot of work too, I would say even to convince them that something had to be done, despite everything I described.”*  *Government professional (2016)*  *“[…] Over and over again, health authorities are approaching us with a sounder vision of what to do at the public health level […]”*  *Government professional (2016)* |
|  | Strategies used by nutritionists to negotiate an agreement & to engage ministerial delegates (21) | *“We must not underestimate the work we did to present the problem and present the solutions we proposed with the scientific rational and very concrete illustrations, because the environments did not speak much to people out there; it was necessary to promote it, a lot. It was necessary to bring very concrete proposals too; create supportive environments for healthy eating, what do you mean? What are you talking to me about? we took very concrete illustrations […] and we also had to prepare presentations […] to present the theoretical concept”.*  *Government professional (2016)* |
| Political strategies | Political strategies of the nutritionists’ coalition (22) | *“… we had the knowledge, and then we reached for ministries and told them for instance ‘maybe we should build bike paths to help us prevent chronic diseases’ and then people looked at us and said ‘we don’t understand’[…] then we told them: ‘Listen, we have a mandate, and it is the Premier who is asking us, we have no choice, we have to do it’ [..] But we had no time to sit with them and then be able to talk […] I think […] we were rushing people a bit, it was not always easy for them because we did not have time to sit and to look at all the implications for everyone...[…]"*  *Government director, 2016*  *“We wanted to impose our own agenda and I do not know to which extent we managed to influence the agenda of the others…”*  *Government professional, 2016*  *“…On the other hand, I think that public health had already prepared the ground with important documents and which served as the basis of the exchanges that we had [in reference to the PTPY]. Then, honestly, I'll tell you there might have been, I'm saying that retrospectively, but there might have been some bias in the composition of the working group, and I'm not saying that necessarily negatively […] They recruited actors who were for prevention, who were, who already had a positive perception of prevention and who had a vision of what prevention could be. So, they gathered around the table people from different sectors, which is perfect, but people who had already identified prevention as a priority [...] I think the people around the table had been carefully chosen for the contribution they could make to this discourse that is to be created on prevention […] So, around the table we only had people who could get along.”*  *Anonymous (2016)* |
|  | Political strategies of other coalitions (25) | *HLPAC - EEAC - “The education [in reference to the Ministry of Education] started a food policy in schools, that also helped us, we managed to work a bit with them, we tried to influence them on the content, it was very difficult because they were very closed […]. They had given [the policy] to people […] from Laval University who worked on the content […]. So they were the ones who were given the mandate to work with the Ministry of Education on policy development.”*  *Government professional (2016)*  *HLPAC - EEAC - “I remember that health [in reference to the Ministry of Health] said basically it's something that we want to put forward as a strong measure in the GAP [in reference to the school food policy]. I remember that […] the Minister of Education at the time, said ‘no , no, we're talking about schools’ he said: ‘ I will develop the policy and it is my ministry which will develop it’”.*  *Government professional (2016)*  *AFAC – EEAC - “At the level of the Ministry of Health, they believed a lot in the positive effect of a tax on junk food and I told them that I did not think that it was an effective intervention when it comes to food, especially that taxing food that is perceived to be less healthy can have a negative impact on the less well-off. The thing to remember is that, unlike smoking, eating is not a choice, and that is what I told them at the time.”*  *Government director (2016)*  *CDAC - EEAC - “Then obviously the government action plan, well, the health ministry in particular, brought together all the ministries and it was a rather long process all the same; we were also consulted, invited to this exercise where we were able, I know that we were part of different exchanges I can’t remember precisely when it was within the process but […], like many other organizations, we can give our opinion on the progress of that, then at some point when it was adopted well obviously everyone was very happy.”*  *Anonymous (2017)*  *CDAC – “At the same time, our president then, who was a member of this working group [in reference to the PTPY], at the conclusion of the recommendations, prompts a challenge: ‘we are willing to put this much money if the government of Québec is willing to put as much’. Then, it was the start of the second phase of partnership with Québec en Forme, from 2007 to 2017, on a partnership with the Government of Québec […] which was part of the process of developing the governmental action plan.”*  *Anonymous (2017)* |
| Influence of strategies on the GAP | Influence of partnerships on the GAP agenda-setting and formulation (23) | *“So, we were very connected, because we were connected with the PWG, with all the PWG partners, we were connected with the Perrault Work Group [in reference to the PTPY] and we were connected with the public health network, so we could channel all these works to the benefit of writing the GAP, so when we started writing the GAP, we did not start from scratch, we already had proposals”*  *Government professional (2016)* |
|  | Influence of the evidence on the GAP content (24) | *“We were very much inspired by the determinants and then the work done in other countries, the recommendations of the WHO and of course the ‘Call to Action’ […] So, we held the workshops which gathered around fifteen people from the public health network to come up with recommendations […] So, we did not start from a blank page when the command arrived.”*  *Government professional (2016)* |
| Policy core beliefs of coalitions (other than EEAC) | Individual responsibility for action & the concept of enabling environments (20) | *“… the famous perception that it is an individual responsibility was still very much rooted […] at the level of our inter-ministerial partners, a lot, a lot; it's a question of individual responsibility: you choose what you eat, you choose to move or not, it's a matter of education, it came back a lot, a lot”.*  *Government professional (2016)*  *“what must be understood is when this concept of enabling environments came out with the GAP, people said ‘what is meant with this?’”*  *Government director (2016)* |
| Policy core Belief (EEAC) | Priority to all the population, not only to children (26) | *“we, from the point of view of public health, the focus exclusively on young people was not necessarily the right priority because it reproduced what we have done in tobacco for a long time; that the prevention of smoking, it is just for the youth, we will educate the youth […], but everyone around will continue to smoke, so it does not transform social norms, if adults do not change their behavior, if there is no transformation of society, thinking that just by educating the youth in an environment where everyone does the opposite, well, the main power of learning is social learning in youth, they will learn, then they will live similarly to how their parents live, their grandparents live, their friends live…”*  *Government director (2016)*  *“You could not deprive promotion and prevention in environments simply by targeting children. Automatically you would reach people, adults, parents, automatically you would reach all your society. […] That's the philosophy of Baum in the new public health […]. That’s what they tell us, that you are going to reach for people where they […] are living, eating, being entertained, studying, working […]”.*  *Government professional (2016)* |
| Negotiations within the policy subsystem | Perception of policy participants of the GAP agenda-setting and formulation (27) | *“…we have experienced a phenomenon that had almost never occurred; that ministries accompanied us, gave us information, they were in back venture, they were taking notes; this was the first-time ministries sat down to listen to what local actors had to say, then for us, it was a really meaningful experience […] And this was the only committee that I worked on, out of three others, where seven ministries were present, ministries that barely communicated with each other earlier… then for us it was an obvious sign of interest. And I think that yes this committee did a fantastic job […].”*  *President provincial organization (2016)* |

##### EEAC, enabling environment advocacy coalition; AFAC, agri-food advocacy coalition; HLPAC, healthy lifestyles promoting advocacy coalition; CDAC, community development advocacy coalition; PWG, provincial workgroup on weight-related problems; NPHD, national public health director; HIV, human immunodeficiency virus; PTPY, provincial task force on prevention in youth; MOH, ministry of health and social services; APHM, annual public health meetings; GAP, governmental action plan; WHO, world health organization

§ Illustrative Quote
