# Supplementary material for: Nutritionists as policy advocates: the case of obesity prevention in Quebec, Canada
Source: Public Health Nutr. 2021 Dec 27;25(7):2011–24. doi: 10.1017/S1368980021004997 (PMC9991765; doi:10.1017/S1368980021004997)
Supplement: Supplementary file 1 [file S1368980021004997sup.zip › S1368980021004997sup003.docx]

| **Table 3.** Provincial Workgroup on Weight-Related Problems in Quebec (PWG)^a^ | |
| --- | --- |
| **Affiliations of PWG members** | **Profession** |
| National Public Health Institute of Quebec | Medical doctor |
| National Public Health Institute of Quebec | Nutritionists and scientific consultants |
| Regional Public Health directorate of Montérégie | Nutritionist |
| Association for Public Health of Quebec | Communication professional |
| Kino-Québec - Sports and Leisure Secretariat | Physical educator |
| Heart and Stroke Foundation of Quebec | Nutritionist |
| Research chair on Obesity, University Laval | Nutritionist and researcher |
| ÉquiLibre (Provincial organism on promoting a positive body image and healthy eating habits) | Nutritionist |
| General Directorate of Public Health, Ministry of Health and Social Services | Nutritionist |
| Department of Kinesiology at the University of Montreal | Kinesiologist and researcher |
| **Collaborators to the PWG** | |
| National Public Health Institute of Quebec | Nutritionists |
| Heart and Stroke Foundation of Quebec | Nutritionist and project leader |
| La Boîte de Comm. | Strategic consultant - Communications and public relations professional |
| Group of collective kitchens of Quebec | Professional specialized in applied social sciences and community economic development |
| Association for Public Health of Quebec | Professional in communications and knowledge mobilization |

^a^The affiliations are based on the PWG memberships in 2005^(23)^. Participation to the PWG was fluid during the period of its activity.
